# Anti‐melanoma differentiation‐associated gene 5 antibody–positive dermatomyositis with possible complication of thrombotic microangiopathy

**DOI:** 10.1111/1346-8138.17004

**Published:** 2023-10-13

**Authors:** Yumi Ito, Saki Takeuchi, Takahisa Tozawa, Satoko Hisada, Yoshihiro Yamada, Masanari Kodera, Masahiro Kobayashi, Mizuho Shirahata, Akihiro Matsubara

**Affiliations:** ^1^ Department of Dermatology Japan Community Health Care Organization Chukyo Hospital Nagoya Japan; ^2^ Department of Respiratory Medicine Japan Community Health Care Organization Chukyo Hospital Nagoya Japan; ^3^ Department of Hematology Japan Community Health Care Organization Chukyo Hospital Nagoya Japan; ^4^ Department of Dermatology Nagoya City University Nagoya Japan

**Keywords:** a disintegrin‐like and metalloprotease with thrombospondin type 1 motifs 13, hematuria, immunosuppressive therapy, psychiatric symptom, thrombocytopenia

## Abstract

This case study illustrates a 63‐year‐old Japanese woman who presented with anti‐melanoma differentiation‐associated gene 5 antibody–positive dermatomyositis. She was administered a therapeutic regimen consisting of corticosteroids, tacrolimus, and cyclophosphamide. However, after a month of treatment, symptoms of confusion and depressive tendencies emerged, followed by the manifestation of hematuria, thrombocytopenia, and fragmented erythrocytes. A disintegrin‐like and metalloprotease with thrombospondin type 1 motifs 13 activity was 45%. Thrombotic microangiopathy was contemplated, yet a definitive diagnosis remained elusive. She died 2 months after admission. Although the occurrence of thrombotic microangiopathy in patients with dermatomyositis is rare, the prognosis is poor, emphasizing the importance of prompt diagnosis and treatment.

## 1 INTRODUCTION

Anti‐melanoma differentiation‐associated gene 5 (anti‐MDA5) antibody–positive dermatomyositis (DM) is frequently associated with the rapid onset of progressive interstitial lung disease. As a result, early and intensive immunosuppressive therapy utilizing glucocorticoids, calcineurin inhibitors, and cyclophosphamide is necessary.^1^

Thrombotic microangiopathy (TMA) is a microvascular occlusive disorder characterized by systemic or intrarenal platelet aggregation, thrombocytopenia, and fragmented erythrocytes.^2^ TMA encompasses thrombotic thrombocytopenic purpura (TTP), hemolytic uremic syndrome (HUS), and TMA that arises secondary to connective tissue disease (CTD), drugs, and other conditions.^2^, ^3^ Numerous patients with acquired idiopathic (ai)‐TTP have a disintegrin‐like and metalloprotease with thrombospondin type 1 motifs 13 (ADAMTS13) inhibitor and reduced ADAMTS13 activity, which cleaves von Willebrand factor (vWF), resulting in unusually large vWF multimers binding to platelets and causing microvascular thrombi.^2^ However, previous reports have shown that relatively few patients with CTD‐TMA have a severe deficiency in ADAMTS13 activity. Moreover, in both ai‐TTP and CTD‐TMA, better therapeutic outcomes were observed when ADAMTS13 activity was significantly deficient.^3^

We report a patient exhibiting psychiatric symptoms and thrombocytopenia during treatment of anti‐MDA5 antibody‐positive DM, which is a possible complication of TMA.

## 2 CASE REPORT

**FIGURE 1:**
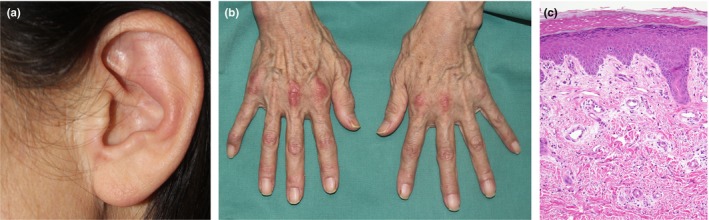
Erythema in the auricles (a) and nail circumference and Gottron sign on hands (b). Skin biopsy from an erythematous area on the dorsal hand showed dermal edema, mild lymphocytic infiltration, and a small amount of mucin deposition (hematoxylin and eosin stain, original magnification ×200) (c).

A 63‐year‐old Japanese woman presented with erythema and dolor in bilateral hands for approximately 1 month. She also complained of arthralgia in the elbow and dyspnea, and could not hold her hands due to pain. She visited a local doctor a week ago, but her symptoms deteriorated, necessitating admission to our hospital. Despite normal oxygen saturation, she manifested dysphagia, dysphonia, and low‐grade pyrexia. She exhibited erythema in the auricles and nail circumference; Gottron sign on both hands, elbows, and knees; reverse Gottron sign on the hands; and grip myalgia in the upper limbs and thighs. Histopathological examination of the dorsal hand erythema revealed edema, mild lymphocytic infiltration, and a small amount of mucin deposition in the dermis (Figure 1). Her laboratory findings exhibited increased myogenic enzymes (aspartate aminotransferase 79 IU/L, lactate dehydrogenase [LDH] 429 IU/L, creatine phosphokinase 254 IU/L), elevated ferritin levels measuring 627 ng/mL, and the presence of positive anti‐MDA5 antibodies. Urinalysis indicated proteinuria at 30> mg/dL and no hematuria.

**FIGURE 2:**
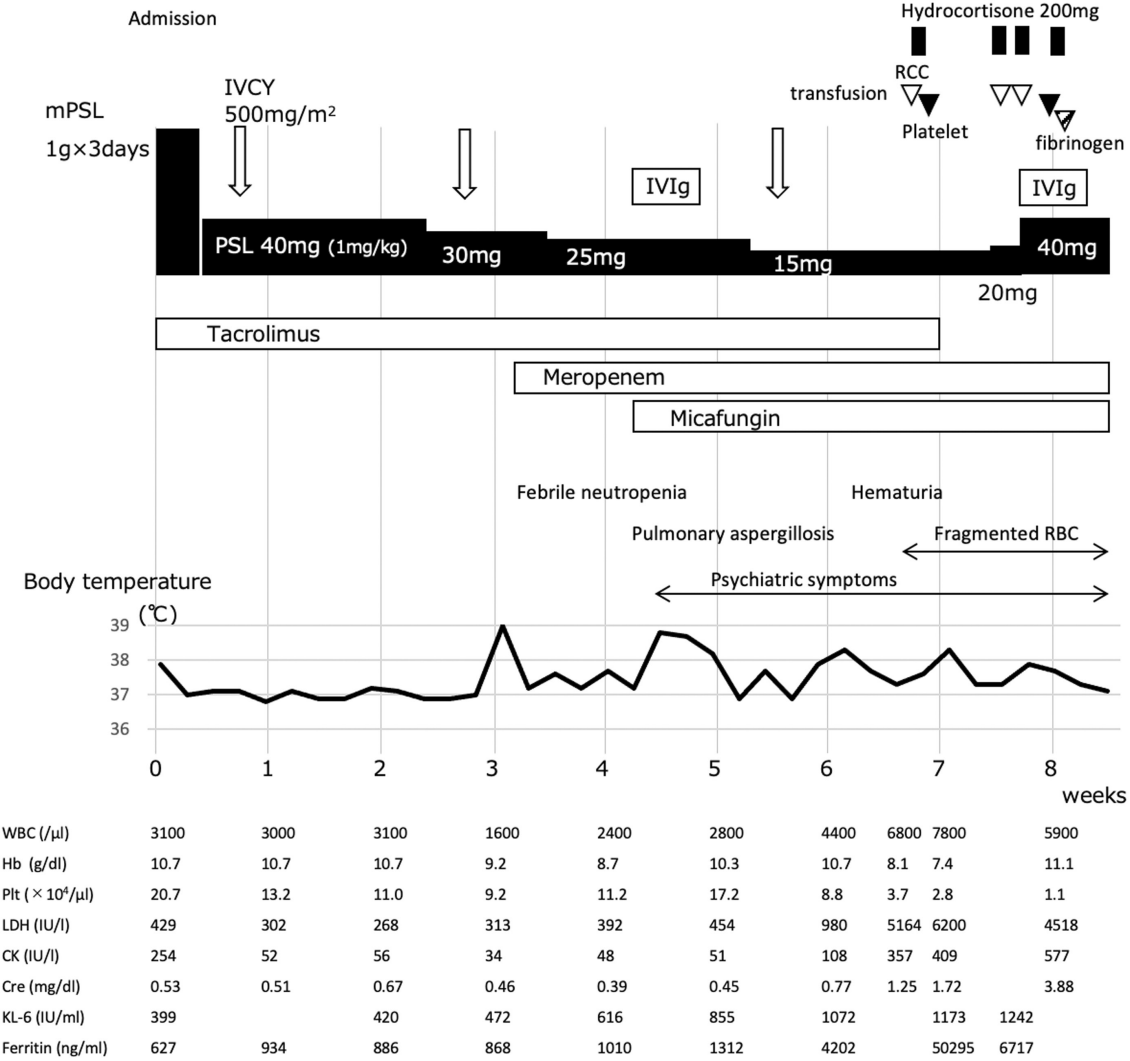
Clinical course. CK, creatine phosphokinase; Cre, creatinine; Hb, hemoglobin; KL‐6, Krebs von den Lungen‐6; LDH, lactate dehydrogenase; Plt, platelet; IVCY, intravenous cyclophosphamide; IVIg, intravenous immunoglobulin; mPSL, methylprednisolone; PSL, prednisolone; RBC, red blood cell; RCC, red cell concentrate; WBC, white blood cell.

Imaging revealed mild interstitial pneumonia, while respiratory function tests indicated a reduction in vital capacity by 48% and carbon monoxide diffusing capacity by 74%. Despite undergoing thoracoabdominal computed tomography (CT), echocardiography, Holter electrocardiography, upper and lower gastrointestinal endoscopy, mammography, and gynecological examination, no abnormalities were detected. She was diagnosed with DM and was expected to experience rapid progression of interstitial pneumonia due to elevated ferritin levels. Consequently, a methylprednisolone pulse of 1 g for 3 days and tacrolimus were initiated on admission (Figure 2). Following the steroid pulse, prednisolone was gradually tapered down from 40 mg and cyclophosphamide 500 mg/m^2^ was administered every 2 weeks. The myogenic enzymes decreased rapidly and within 1 to 2 weeks of admission, the rash became milder, and symptoms such as arthralgia, muscle weakness, respiratory distress, dysphagia, and dysphonia disappeared.

She developed febrile neutropenia and pulmonary aspergillosis at approximately 3 and 4 weeks into her hospitalization, respectively. Treatment was administered with meropenem and micafungin, respectively, providing mild relief. Despite receiving intravenous immunoglobulin (IVIg) therapy, ferritin and Krebs von den Lungen‐6 levels remained elevated. Around the 32nd day of hospitalization, she exhibited symptoms of mental confusion, including an inability to maintain a sense of order in her surroundings, forgetfulness, and depressive tendencies. Head imaging was normal and treatment with antidepressants proved ineffective. Although steroid psychosis was suspected, her psychiatric symptoms did not improve after reducing the prednisolone dosage. On day 45 of hospitalization, she presented with hematuria, anemia. On day 48, fragmented erythrocytes were observed and platelets decreased rapidly from 9.9 × 10^4^/μL to 3.7 × 10^4^/μL. Coagulation tests were normal, and cytomegalovirus antigen was negative. Pretransfusion testing revealed a positive direct Coombs test and autoimmune hemolytic anemia was considered, but bilirubin and haptoglobin levels were within normal range. Immune thrombocytopenia was also considered due to elevated platelet‐related IgG. Her overall condition deteriorated, and bone marrow examination could not be performed. Consulting with a hematologist, red blood cells and platelets were transfused under hydrocortisone administration. ADAMTS13 activity was recorded at 45%, and the ADAMTS13 inhibitor tested negative. Rapid increases in tacrolimus blood levels, from approximately 10 ng/mL until day 44 of hospitalization to 49.8 ng/mL on day 49, led to the discontinuation of tacrolimus administration.

Thrombocytopenia, anemia, fragmented erythrocytes, and psychiatric symptoms led to a suspicion of TMA. Sputum did not detect *Streptococcus pneumoniae*. Although transient episodes of hematochezia and diarrhea were observed, fecal specimens yielded no evidence of bacterial colonization, renal function was preserved, and HUS was excluded. Consultation was sought for plasma exchange therapy, but the nephrologist opined that disseminated intravascular coagulation (DIC) should be considered given normal hemolytic markers. Fibrinogen/fibrin degradation products (FDPs) registered an elevation to 40 μg/mL, thus reaching a score of 6 points according to the diagnostic criteria for acute DIC established by the Japanese Association for Acute Medicine in 2005. In response to her cytopenia, blood transfusions were administered, accompanied by IVIg and fibrinogen supplementation. However, her renal function gradually declined with eventual observation of elevated bilirubin and decreased haptoglobin. Further treatment was being considered when her state of consciousness deteriorated, culminating in convulsions and death 2 months after admission. Postmortem CT revealed pulmonary edema with no apparent head changes.

## 3 DISCUSSION

In our case, psychiatric manifestations anteceded, with hematological abnormalities. Kalish et al. documented a case of TTP in a 35‐year‐old man who experienced a cerebral infarction, followed by gastrointestinal hemorrhage, hematuria, thrombocytopenia, anemia, heightened LDH levels, and fragmented erythrocytes 13 days later. In this case, a stored plasma sample taken on the initial day of admission exhibited diminished ADAMTS13 activity and ADAMTS13 inhibitor negative. The authors deliberated on the plausibility that a thrombotic event may be focal in the early stages of TTP, preceding the development of hematological signs.^4^

In our case, DIC was also considered due to elevated FDP. Bone marrow examination was not performed, but the most likely possibility of TMA was considered due to the presence of fragmented erythrocytes, psychiatric symptoms, and eventually elevated bilirubin and decreased haptoglobin. Coombs test is negative in TMA but was positive in this case. This may be due to antibody contamination by IVIg.^5^ Platelet‐associated IgG is also reported to be elevated in TTP.^6^ Potential factors that may contribute to the development of TMA encompass DM, febrile neutropenia, pulmonary aspergillosis, and tacrolimus.^2^ Assessment of complement regulators was omitted.

In ai‐TTP, the activity of ADAMTS13 is substantially decreased, leading to the failure of vWF cleavage and the occurrence of microvascular thrombosis. However, a study by Matsuyama et al. utilizing Nara Medical University's nationwide database of patients with TMA in Japan found that fewer patients with CTD‐TMA had severely reduced ADAMTS13 activity compared with those with ai‐TTP. Among the 64 patients with ai‐TTP from March 2006 to April 2008, 45 patients (70.3%) had severely reduced ADAMTS13 activity to <0.5% of that of healthy patients, 15 patients (23.4%) had ADAMTS13 activity between 0.5% and 25%, and four patients (6.3%) had ADAMTS13 activity >25%. In contrast, among the 127 patients with CTD‐TMA from 1997 to 2006, 21 patients (16.5%) had <0.5%, 40 patients (31.5%) had between 0.5% and 25%, and 66 patients (52.0%) had >25%. The group with severely reduced ADAMTS13 activity had a trend toward less frequent renal involvement, higher remission rates, and lower mortality rates than the other groups.^3^

In cases of TMA or TTP with PM/DM, a total of 15 cases, including our own, were reported to have their ADAMTS13 activity measured (Table 1). However, only one case demonstrated an activity reduction of <10%, consistent with the diagnosis of TTP.^7^ In our case, during the initial treatment of DM, psychiatric symptoms and thrombocytopenia emerged, with potential links to the underlying collagen disorder's symptoms, medication side effects, and the development of new complications. Furthermore, ADAMTS13 activity was relatively well preserved at 45%, and ADAMTS13 inhibitor tested negative, making the diagnosis difficult and the prognosis unfavorable.

**TABLE 1:** ADAMTS13 levels in cases of thrombotic microangiopathy or thrombotic thrombocytopenic purpura with polymyositis/dermatomyositis.

	Authors	Age	Sex	Type of myositis	ADAMTS13		Onset of TMA or TTP		Outcome
					Activity	Inhibitor	From onset of PM/DM (from start of treatment of PM/DM)	PM/DM state	
1	Sugimoto, et al (2007)	50	F	DM	45%	ND	(1 year 4 months)	Remission phase	Alive
2	Fujisawa, et al (2008)^a^	43	F	DM/SSc	20%	−	2 years 8 months	Remission phase	Alive
3	Maruyama, et al (2008)^a^	66	M	DM	37%	ND	3 months (half month)	Under initial treatment	Alive
4	Iwagami, et al (2011)	70	F	PM/SSc	86%	−	6 years	Remission phase	Alive
5	Bader‐Meunier, et al (2012)	7	M	JDM	Normal	ND	3 weeks	Simultaneous onset	Alive
6	Tachikawa, Ichikawa (2014)^a^	61	F	DM	50.9%	−	3.5 months (43 days)	Under initial treatment	Dead
7	Soejima, et al (2016)^a^	65	F	DM	Normal	ND	1 month (14 days)	Under initial treatment	Alive
8	Tamura, et al (2017)^a^	65	F	PM/RA	50%	−	17 years	Remission phase	Alive
9	Yamada, et al (2018)	70	M	DM	Not detected	+	6 months	Relapse phase	Dead
10	Yamada, et al (2018)	65	F	DM	29.1%	+−	10 years	Relapse phase	Dead
11	Hayashi, et al (2019)	28	F	DM	17.8%	−	1 month (half month)	Under initial treatment	Alive
12	Pérez, et al (2019)	50	M	DM/SSc	Normal	ND	2 weeks	Simultaneous onset	Alive
13	Gullapalli, et al (2020)	12	F	JDM	64%	ND	7 weeks (5 weeks)	Relapse phase	Alive
14	Yamamoto, et al (2021)	56	F	DM	Normal	−	5.5 months (half month)	Under initial treatment	Dead
15	Our case	63	F	DM	45%	−	2 months (1 month)	Under initial treatment	Dead

Abbreviations: ADAMTS13, a disintegrin‐like and metalloprotease with thrombospondin type 1 motifs 13; DM, dermatomyositis; JDM, juvenile dermatomyositis; ND, no data; PM, polymyositis; RA, rheumatoid arthritis; SSc, systemic sclerosis; TMA, thrombotic microangiopathy; TTP, thrombotic thrombocytopenic purpura.

^a^ Japanese article.

CTD‐TMA has a poor prognosis and may contain a different mechanism than ai‐TTP. Further clarification of the pathogenesis and establishment of treatment methods are desirable.

## CONFLICT OF INTEREST STATEMENT

None declared.

## References

[1] McPherson M , Economidou S , Liampas A , Zis P , Parperis K . Management of MDA‐5 antibody positive clinically amyopathic dermatomyositis associated interstitial lung disease: a systematic review. Semin Arthr Rheum. 2022;53:1–13. 10.1016/j.semarthrit.2022.151959 35134633

[2] Moake JL . Thrombotic microangiopathies. N Engl J Med. 2002;347:589–600. 12192020 10.1056/NEJMra020528

[3] Matsuyama T , Kuwana M , Matsumoto M , Isonishi A , Inokuma S , Fujimura Y . Heterogeneous pathogenic processes of thrombotic microangiopathies in patients with connective tissue disease. Thromb Haemost. 2009;102:371–378. 19652889 10.1160/TH08-12-0825

[4] Kalish Y , Rottenstreich A , Rund D , Hochberg‐Klein S . Atypical presentations of thrombotic thrombocytopenic purpura: a diagnostic role for ADAMTS13. J Thromb Thrombolysis. 2016;42:155–160. 26867546 10.1007/s11239-016-1342-7

[5] Parker V , Tormey CA . The direct antiglobulin test: indications, interpretation, and pitfalls. Arch Pathol Lab Med. 2017;141:305–310. 28134589 10.5858/arpa.2015-0444-RS

[6] Kelton JG , Powers PJ , Carter CJ . A prospective study of the usefulness of the measurement of platelet‐associated IgG for the diagnosis of idiopathic thrombocytopenic purpura. Blood. 1982;60:1050–1053. 6889450

[7] Yamada S , Yamashita H , Nakano M , Hatano H , Sasaki T , Takahashi Y , et al. Thrombotic microangiopathy with polymyositis/dermatomyositis: three case reports and a literature review. Intern Med. 2018;57:2259–2265. 30068898 10.2169/internalmedicine.0512-17 PMC6120848

